# Titanate Nanowires as One-Dimensional Hot Spot Generators for Broadband Au–TiO_2_ Photocatalysis

**DOI:** 10.3390/nano9070990

**Published:** 2019-07-09

**Authors:** Yoel Negrín-Montecelo, Martín Testa-Anta, Laura Marín-Caba, Moisés Pérez-Lorenzo, Verónica Salgueiriño, Miguel A. Correa-Duarte, Miguel Comesaña-Hermo

**Affiliations:** 1 Department of Physical Chemistry, Biomedical Research Center (CINBIO), Southern Galicia Institute of Health Research (IISGS), and Biomedical Research Networking Center for Mental Health (CIBERSAM), Universidade de Vigo, 36310 Vigo, Spain; 2 Departamento de Física Aplicada, Universidade de Vigo, 36310 Vigo, Spain; 3 Université de Paris, ITODYS, CNRS, UMR 7086, 15 rue J-A de Baïf, F-75013 Paris, France

**Keywords:** titanates, photocatalysis, plasmonic hot spots, hierarchical assembly

## Abstract

Metal–semiconductor nanocomposites have become interesting materials for the development of new photocatalytic hybrids. Along these lines, plasmonic nanoparticles have proven to be particularly efficient photosensitizers due to their ability to transfer plasmonic hot electrons onto large bandgap semiconductors such as TiO_2_, thus extending the activity of the latter into a broader range of the electromagnetic spectrum. The extent of this photosensitization process can be substantially enhanced in those geometries in which high electromagnetic fields are created at the metal–semiconductor interface. In this manner, the formation of plasmonic hot spots can be used as a versatile tool to engineer the photosensitization process in this family of hybrid materials. Herein, we introduce the use of titanate nanowires as ideal substrates for the assembly of Au nanorods and TiO_2_ nanoparticles, leading to the formation of robust hybrids with improved photocatalytic properties. Our approach shows that the correct choice of the individual units together with their rational assembly are of paramount importance in the development of complex nanostructures with advanced functionalities.

## 1. Introduction

The combination of nanomaterials with different compositions and functionalities has proven to be a promising method to unravel new features and promote the appearance of synergistic effects at the nanoscale. In this manner, theranostic platforms, heterogeneous catalysts or microelectronic components exemplify the interest in the design of nanostructured composites with a higher degree of complexity [1,2,3]. Among the large number of nanocomposites under study, the formulation of hybrid photocatalysts with broadband activities has been at the center of many synthetic efforts. The main interest of this family of materials resides in the synergistic interaction between a large bandgap semiconductor such as TiO_2_ (bandgap energy: E_BG_ ≅ 3.0–3.5 eV) and a photosensitizer, leading to the formation of hybrid materials in which the photocatalytic properties of the former can be expanded to a broader range of excitation wavelengths (commonly, in the visible and near-infrared (NIR) ranges of the electromagnetic spectrum). Plasmonic nanoparticles (NPs) are exceptional photosensitizers given their ability to efficiently harvest visible and NIR photons [4]. Upon plasmonic excitation, part of this energy is transferred to conduction electrons, leading to the formation of a population of “hot” carriers that can be subsequently transferred to the conduction band of a nearby semiconductor [5]. In order to attain an efficient hot electron injection from the plasmonic component to the acceptor, two important characteristics have to be fulfilled: (a) the correct band alignment between the Fermi level on the metal and the conduction band of the semiconductor, and (b) the formation of a Schottky barrier between both components [6].

Hybrids composed of the combination of Au and TiO_2_ nanoparticles have proven to be particularly valuable because the low energy difference between the Fermi level of the plasmonic metal and the conduction band of the semiconductor (~1 eV) allows for an efficient electron transfer in those cases in which a Schottky barrier is created between them [6]. Moreover, Au NPs are highly stable and can be easily synthesized in a vast variety of anisotropic shapes, a characteristic that plays a relevant role in the photosensitization process. Indeed, previous reports have shown that certain metal morphologies can favor the formation of hot spots, understood as regions at the metallic surface in which the intensity of the local electromagnetic field can be enhanced by several orders of magnitude [6,7,8]. Accordingly, the presence of these plasmonic hot spots at the Schottky barrier maximizes the electron injection and, ultimately, the photosensitization of the semiconductor. In a similar way, the narrow gaps created through the controlled assembly of plasmonic objects can result in a further enhancement effect [9,10]. These examples show that a rational design of the hybrid photocatalyst and hence, the specific combination of the different components, can lead to better physical interactions, with consequent improved photocatalytic capabilities.

**Scheme 1:**
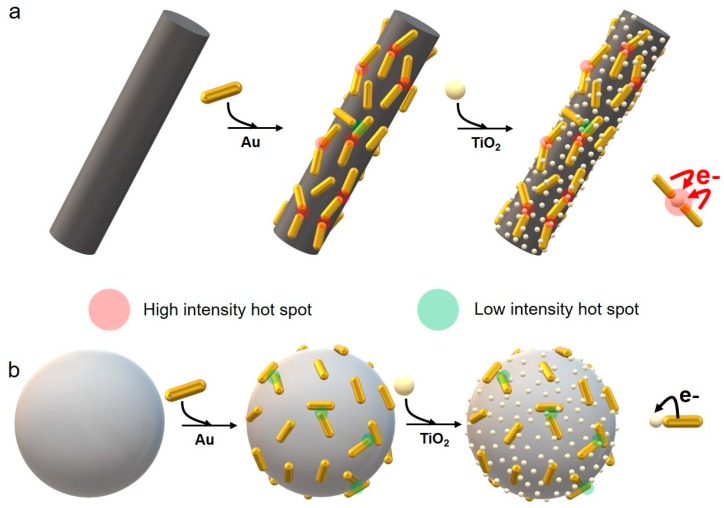
Illustration depicting the formation of a hybrid composite in which the use of Ti NWs as a template favors the tip-to-tip coupling of Au NRs (**a**) compared to that found on a spherical silica support in which a random distribution is observed (**b**).

In the present work, we introduce the use of one-dimensional titanate nanostructures as outstanding substrates for the development of hybrid Au-TiO_2_ photocatalysts with improved features. Nanostructured titanates have emerged as interesting materials for a large number of applications, ranging from H_2_ storage to the fabrication of piezoelectric components or the catalyzed removal of pollutants [11,12,13]. Along these lines, we have previously taken advantage of titanate nanotubes as efficient photocatalysts for the selective photodegradation of organic contaminants in complex environments [14]. In that work, the photo-induced growth of spherical Au NPs onto the surface of the nanotubes was shown to be an efficient means to obtain a broadband activity. Nevertheless, the hollow structure and small diameter (8 nm) of these objects result in poor mechanical properties, thus making these substrates less reliable for the deposition of inorganic nanomaterials. In this study, we replaced those hollow nanostructures with titanate nanowires (Ti NWs), a material that presents improved crystallinity and larger diameters, while keeping extremely high aspect ratios with lengths above the micrometer scale. Such characteristics confer titanate nanowires with enhanced mechanical capabilities, thus allowing the adsorption of pre-synthesized objects and a more versatile control over the final properties of the composite material. Accordingly, we have taken advantage of these remarkable features in order to perform a rational assembly of Au nanorods (NRs) and TiO_2_ NPs. In this case, the advantages associated with the use of titanate nanowires as substrates are manifold: (i) the high aspect ratio of these structures allows for a preferential one-dimensional assembly of the nanorods, hence leading to a tip-to-tip configuration in which the plasmonic coupling results in the formation of hot spots (Scheme 1) [15]; (ii) the use of a nanostructured template gives rise to a homogeneous and controlled distribution of the plasmonic objects and the TiO_2_ NPs, hence facilitating the control over critical parameters such as their physical coupling and the metal/semiconductor ratio; (iii) the surface area of Ti NWs is larger than that offered by other colloidal substrates, and (iv) titanate nanowires can be easily isolated from solution by centrifugation, ensuring a straightforward recovery and reuse.

## 2. Materials and Methods

### 2.1. Materials

Tetrachloroauric acid (HAuCl_4_·3H_2_O), hexadecyltrimethylammonium bromide (CTAB), sodium borohydride (NaBH_4_), silver nitrate (AgNO_3_), hydrochloric acid (HCl), l-ascorbic acid (AA), sodium hydroxide (NaOH) pellets, poly(allylamine hydrochloride) (PAH), sodium chloride (NaCl), poly(styrenesulfonate) (PSS) and sodium citrate (Na_3_C_6_H_5_O_7_), were purchased from Sigma-Aldrich (St. Louis, MO, USA). TiO_2_ NPs with a diameter of 5 nm were purchased from Nanoamor (Katy, TX, USA). Milli-Q water with a resistivity higher than 18.2 MΩ·cm was used for all the preparations.

### 2.2. Synthesis of Ti NWs and Subsequent PAH Coating

Ti NWs (with a mean length >20 μm and a mean diameter of 57 nm) were synthesized following a previously reported procedure [16]. Briefly, 0.2 g of TiO_2_ NPs (5 nm) were mixed with 20 mL of an aqueous solution of NaOH (8 M) and stirred for 10 min at room temperature. Then, the mixture was placed in a Teflon-lined stainless steel autoclave and introduced inside an oven at 240 °C, where the hydrothermal treatment proceeded for 5 h. After that, the white precipitate was washed at least five times following centrifugation–redispersion cycles (5000 rpm, 20 min) until the pH reached neutrality. The solid obtained was finally redispersed in 20 mL of water. The Ti NWs synthetized had a negative surface charge (−37.2 ± 1 mV) and were subsequently coated with a layer of a positively charged polyelectrolyte (PAH). For this purpose, PAH was dissolved in a 0.5 M NaCl solution (pH 5) with a final polymer concentration of 1 mg/mL. Then, 100 mL of the positively charged PAH solution were added to the Ti NWs (20 mg) and stirred at room temperature for 30 min. The excess of reagents was removed by three centrifugation–redispersion cycles with water (6000 rpm, 20 min).

### 2.3. Synthesis of Au NRs and PSS Coating

Au NRs with a longitudinal plasmon mode centered at 873 nm were synthesized by the seed-mediated growth method as described elsewhere [17]. The dimensions obtained from transmission electron microscopy (TEM) pictures were 55 ± 5 nm in length and 11 ± 1 nm in thickness. The Au NRs were subsequently coated with a layer of a negatively charged polyelectrolyte (PSS) in order to proceed to the deposition onto the positively charged Ti NWs. This functionalization has been previously reported in the literature [18].

### 2.4. Synthesis of Spherical Au NPs

We synthesized 45 ± 4 nm spherical Au NPs following a previously published protocol that is based on the reduction of HAuCl_4_ onto preformed Au NPs, using citrate as stabilizer and reducing agent [19]. The citrate-stabilized spherical Au NPs obtained possessed a negative surface charge and were used as-synthesized.

### 2.5. Assembly of Au NRs@PSS with the Functionalized Ti NWs

We added 5, 10 and 20 mL of Au NRs@PSS (0.5 mM) to 10 mg of functionalized Ti NWs. The mixture was stirred at room temperature for 3 h and washed by three centrifugation–redispersion cycles with water (6000 rpm, 20 min). In all cases, the products were finally redispersed in 10 mL of water. We proceeded with the assembly protocol of the spherical Au NPs coated with citrate molecules in a similar fashion, adding the same amounts of metals (2.5, 5 and 10 μmol).

### 2.6. Deposition of TiO_2_

We sonicated 25 mg of TiO_2_ (5 nm) redispersed in 50 mL of a sodium citrate solution (2.5 mM) for 1 h with an ultrasonication tip. The aggregated particles were removed by centrifugation (3500 rpm, 10 min). Then, 10 mL of the Ti NWs@Au solution coated with an extra layer of PAH were added to the solution of TiO_2_ NPs and stirred at room temperature for 60 min. The excess of TiO_2_ was removed by three centrifugation–redispersion cycles (6000 rpm, 20 min) and for each sample the product was redispersed in 10 mL of water. Au/TiO_2_ molar ratios of 0.023, 0.059 and 0.1 were obtained by inductively coupled plasma mass spectrometry (ICP-MS) for the three different amounts of metal NPs added (the amount of TiO_2_ NPs was kept constant throughout the entire set of experiments).

### 2.7. Photocatalytic Study

In order to evaluate the photocatalytic activity of the synthetized composites, we performed the degradation of rhodamine B (RhB) in water as a model reaction under light irradiation using a 300 W Xe lamp in a LOT-Quantum Design solar simulator (350−2400 nm). The study was carried out in a 20 mL aqueous solution with a RhB concentration of 1 × 10^−5^ M and 4 mg of hybrid photocatalyst. The mixtures were kept at a controlled temperature (25 °C) and stirred for 1 h in the dark to blend well and provide the necessary time for the adsorption–desorption equilibrium to take place before irradiation, leading to a small loss of 2−3% of the original absorption value of RhB. Subsequently, aliquots of 3 mL were taken within 15 min intervals under irradiation with the solar simulator, allowing the quantification of the photocatalytic process by monitoring the decreasing adsorption of the dye in solution.

### 2.8. Structural and Chemical Characterization

TEM images were obtained using a JEOL JEM 1010 transmission electron microscope (Tokyo, Japan) operating at an acceleration voltage of 100 kV. The degree of alignment of the Au nanorods onto the Ti NWs was measured by using the TEM images. To do so, the Au NRs that follow the axis of the nanowires with a maximum mismatch of ±30° were considered as aligned, whereas the remainder were considered as randomly oriented. In total, more than 500 Au NRs were counted in order to obtain data of statistic value. UV-vis-NIR spectra were obtained with a Hewlett-Packard HP8453 spectrophotometer (CA, USA). Physisorption studies were carried out with N_2_ at 77 K using a Belsorp-max apparatus from MicrotracBEL Corporation (Osaka, Japan). Before being analyzed, the samples were outgassed at 423 K for 12 h under a pressure of 0.1 Pa. The BET processing was carried out in the relative pressure range of 0.05–0.25. The X-ray diffraction (XRD) pattern was collected from the powdered sample on a PANalytical X’Pert Pro diffractometer (Spectris, Egham, United Kingdom), using Cu Kα radiation in a 2θ angular range between 5−90° (continuous scan mode, step = 0.02°, 4 s/step). The refinement of the experimental data was performed using the Le Bail method by means of the software Rietica. Raman spectra were obtained with a Renishaw in a Via Reflex Raman microscope (Wotton-under-Edge, United Kingdom). Experiments were conducted at room temperature using three different lasers as excitation sources: a 532 nm frequency doubled Nd:YAG/Nd:YVO_4_ diode, a 633 nm He–Ne laser, and a 785 nm NIR diode laser. The laser beam was focused onto the sample by a 20x objective, and the spectra were registered during variable acquisition times (either 60 or 120 s) over one accumulation, after applying different laser powers (ranging between 0.37−35.2 mW).

## 3. Results and Discussion

**Figure 1:**
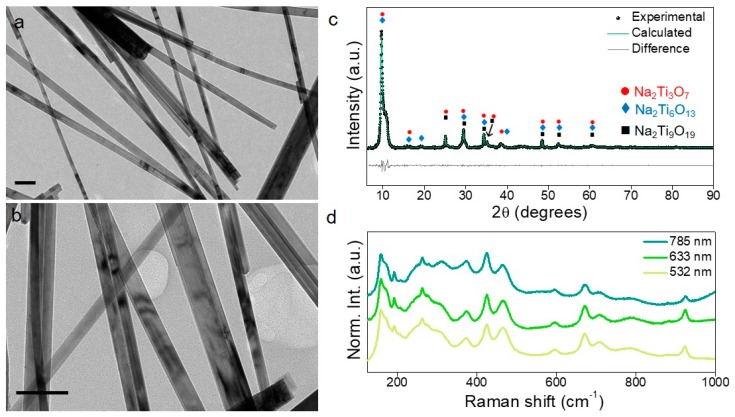
Characterization of the Ti NWs: (**a**,**b**) TEM images of the as-synthesized wires (scale bars represent 200 nm), (**c**) powder XRD pattern, and (**d**) room temperature Raman spectra registered with three different excitation wavelengths.

The synthesis of Ti NWs has been previously reported in the literature [16]. In the present work, TiO_2_ NPs (5 nm) were subjected to a hydrothermal treatment at 240 °C for 5 h in the presence of an 8 M NaOH solution (for more details see the Materials and Methods section). This procedure, based on a collaborative oriented attachment–Ostwald ripening (OA–OR) mechanism leads to the formation of an intermediate nanotube morphology that subsequently evolves towards the more thermodynamically stable nanowire shape. Even though the as-synthesized nanowires present a relatively polydisperse diameter (Figure 1a,b and Figure S1), TEM images show their high shape anisotropy, thus giving rise to aspect ratios as high as ~500 and the absence of other morphologies in the reaction medium. Moreover, Brunauer–Emmett–Teller (BET) analyses show that the as-synthesized Ti NWs have a specific surface area of 89 m^2^/g. This value is very similar to that presented in the literature, being related to the presence of remnant pores in the inner structure of the nanowires [16]. Importantly, the nano-objects presented herein are stable and no morphological defects or degradation under extended sonication treatments were detected. All these characteristics highlight the suitability of these nanostructures as substrates towards the formation of hybrid photocatalysts.

The composition and crystalline structure of the as-synthesized nanowires were assessed by powder X-ray diffraction (XRD) and Raman spectroscopy. Figure 1c includes the XRD pattern of the powdered sample, indicating a strong (100) preferred orientation (diffraction peak at 9.6°) primarily stemming from the unidirectional anisotropy of the nanowires. Despite the presence of relatively broad and asymmetric diffraction peaks, the XRD pattern can be indexed to the sodium trititanate phase (Na_2_Ti_3_O_7_), which crystallizes in a monoclinic system with the space group P21/m. Compared to the bulk and pristine material, significant shifts were observed for the main reflections. Recalling the layered structure of this phase, the shift of the (100) reflection towards lower 2θ values clearly points to an increase in the interlayer distance, suggesting the presence of water molecules at these interlamellar spaces. The shifts observed for the remaining peaks can be associated with slight discrepancies in the Na_2_Ti_3_O_7_ stoichiometry, presumably due to a Na^+^/H^+^ exchange during the manipulation or storage of the nanowires in water [20,21,22]. Indeed, chemical analysis obtained by means of ICP-MS and X-ray fluorescence experiments led to the chemical formula Na_0.915_H_1.085_Ti_3_O_7_·nH_2_O as the main crystalline phase of these structures.

Although the XRD pattern can be mainly attributed to sodium trititanate, small amounts of sodium hexatitanate (Na_2_Ti_6_O_13_) and nonatitanate (Na_2_Ti_9_O_19_) were also found to be present as secondary phases (marked by blue diamonds and black squares in Figure 1c, respectively). Taking into account the presence of these three phases, a refinement of the experimental data was performed by means of the Le Bail method. The calculated lattice parameters for Na_2_Ti_3_O_7_ (see Table S1 for more details) revealed a unit cell expansion along the a- and c-directions (around 1.2% and 1.7%, respectively) with respect to the bulk material. This fact is consistent with the Na_0.915_H_1.085_Ti_3_O_7_·nH_2_O composition mentioned before. Indeed, a mixture of the Na_2_Ti_3_O_7_ and H_2_Ti_3_O_7_ phases would not imply a priori any change in the lattice parameters. On the other hand, an intermediate phase as a result of a Na^+^/H^+^ exchange can account for these variations and the subsequent shift of the trititanate diffraction peaks.

Further insight into the chemical nature and crystalline structure of the sodium titanate nanowires can be achieved by means of Raman spectroscopy. In general, metal-containing titanates led to sharp and very distinctive Raman peaks [23], thus providing a valuable fingerprint for the identification of the different phases that may be present in the system. Bearing in mind that the corresponding XRD pattern reveals the Na_2_Ti_3_O_7_ as the main phase (space group P21/m), and that a sodium trititanate primitive cell (*Z* = 2) comprises 24 atoms (all of them located at the 2e Wyckoff position), group theory predicts the following optical phonon modes in the center of the first Brillouin zone:

(1) $$\Gamma_{opt}=24A_{g}+11A_{u}+12B_{g}+22B_{u}$$

among which, 33 are IR active ($11A_{u}+22B_{u}$) and 36 Raman active ($24A_{g}+12B_{g}$).

The Raman spectra of the sodium titanate nanowires was registered at room temperature using three different excitation wavelengths: 532, 633 and 785 nm (Figure 1d). As expected, a large number of vibrational modes could be observed in the Raman spectrum, with the main features centered at 160, 172, 193, 250, 263, 279, 312, 374, 426, 466, 595, 671, 709, 786, 880 and 924 cm^−1^. Although a previous study has addressed the assignment of each phonon symmetry (*Ag* or *B_g_*) [24], the use of unpolarized Raman measurements together with the proximity in frequency of the different phonon modes prevent us from carrying out an accurate symmetry indexation. Except for the peaks at 172 and 263 cm^‒1^ (attributable to Na_2_Ti_6_O_13_ and H_2_Ti_3_O_7_, respectively), all the remaining Raman peaks can be assigned to Na_2_Ti_3_O_7_ and are in good agreement with the values reported in the literature for this phase [25,26]. It is generally agreed that the modes occurring below 500 cm^‒1^ mostly originate from Na−O−Ti vibrations, whereas those in the 600−800 cm^‒1^ region arise from Ti–O vibrations in edge-shared and corner-shared TiO_6_ octahedra [20,26]. The two vibrations observed in the 800−950 cm^‒1^ region account for short Ti−O bonds of low coordination. Namely, the mode at 880 cm^‒1^ is ascribed to a short Ti−O stretching involving a terminal oxygen atom (i.e., coordinated to just one titanium atom), and the highest frequency mode at 924 cm^‒1^ to a short Ti−O stretching in distorted TiO_6_ octahedra [20,27]. In contrast to other studies in the literature, the spectra included in Figure 1d show that the Raman peak at 880 cm^‒1^ displays a much lower intensity than that found at 924 cm^‒1^. This observation suggests a smaller number of terminal oxygen atoms in the crystal structure of Na_2_Ti_3_O_7_, which corroborates the presence of Na_2_Ti_6_O_13_. Indeed, since the tunnel structure of sodium hexatitanate displays no terminal oxygen atoms (all oxygen atoms at the surface are linearly coordinated to two titanium atoms) [27], the presence of this phase would hinder the vibrational mode at 880 cm^‒1^. Regarding the peak observed at 263 cm^‒1^, the presence of H_2_Ti_3_O_7_ could be considered. However, taking into account that no other distinctive features of this phase are observed and that the low frequency modes in the Na_2_Ti_3_O_7_ Raman spectrum essentially imply Na−O−Ti vibrations, the presence of this peak may be the consequence of the already mentioned partial Na^+^/H^+^ exchange, as suggested by the XRD pattern (*vide supra*). Despite the XRD results pointing to the additional presence of Na_2_Ti_9_O_19_, no direct evidence of this phase can be found in the Raman spectra. Similar observations have been made previously, such that discernibility between different sodium titanates (Na_2_Ti*_n_*O_2*n*+1_, *n* = 3, 4, 9) could not be achieved by means of Raman spectroscopy [25,26]. Certainly, these similarities in the Raman spectrum of the Na_2_Ti*_n_*O_2*n*+1_ phases stem from the fact that all *Bg* symmetry modes are related to displacements along the [010] direction [24] and hence, are barely dependent on the n value, which just represents the number of TiO_6_ octahedra in the {Ti*_n_*O_2*n*+1_}^2‒^ ribbons that make up the titanate lamellar structure.

As mentioned before, the Raman spectrum from the sodium titanate sample were registered using three different excitation wavelengths (Figure 1d). Owing to the different penetration depth of these laser sources, the possibility of local phase segregation can be readily assessed [28]. Aside from the better resolution of the mode at 312 cm^‒1^ under the 785 nm laser, the three Raman spectra are virtually identical, and the spectra registered from different spots in the sample led to the same results (see Figure S2d), thereby underlining the homogeneity of the sample presented herein. Additionally, aiming to rule out a potential phase transition due to the energy input provided by the laser source, the Raman spectrum was also registered as a function of the laser power for each excitation wavelength (as shown in Figure S2a–c). Besides the better signal-to-noise ratio at increased laser power, the resulting spectra reflect no changes in the vibrational modes throughout the whole range considered (from 0.4 to 35 mW). This fact highlights the high thermal stability of the sample, which does not undergo transition to Na_2_Ti_6_O_13_ as it has been reported in other studies [20,25,27].

**Figure 2:**
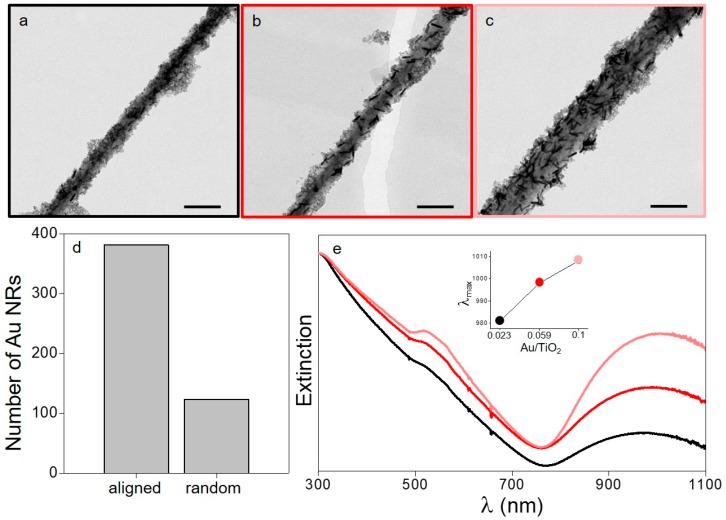
Structural and optical characterization of the titanate nanowires functionalized with Au NRs and TiO_2_ NPs: (**a**–**c**) TEM images with the different Au/TiO_2_ molar ratios: 0.023, 0.059 and 0.1, respectively (the scale bars represent 200 nm), (**d**) number of aligned and randomly oriented Au NRs observed in the samples (the ratio remains stable for the three different amounts of Au NRs introduced), and (**e**) extinction spectra of the three samples with different Au/TiO_2_ molar ratios: 0.023 (black), 0.059 (red) and 0.1 (pink) (the inset shows the shift in the longitudinal plasmon band with respect to the increase in concentration of plasmonic nano-objects).

As previously stated, the Ti NWs presented herein have been used as templates for the controlled deposition of plasmonic objects and small (~5 nm) TiO_2_ NPs, aiming at the formation of a hybrid photocatalyst with enhanced catalytic features. To this end, a “layer-by-layer” process was conducted in order to obtain a sequential adsorption of the different components [29], leading to the formation of reproducible interfaces between the plasmonic photosensitizer and the large bandgap semiconductor, while providing long term colloidal stability to the system. This approach has been previously used by us to attach plasmonic and semiconductor NPs onto colloidal substrates such as silica or poly(N-isopropylacrylamide) (pNIPAM) sub-micrometric spheres [6,10]. In both cases, the homogeneous distribution of both components and the regular interfaces created between them result in the ideal scenario to study hot electron injection mechanisms. In the present case, the use of an anisotropic template allowed us to induce a preferential plasmonic coupling between the Au NRs by means of a tip-to-tip configuration [15]. The first step of the assembly process consists of the electrostatic adsorption of the negatively charged nanorods (or nanospheres) onto the positively charged Ti NWs. Subsequently, the adsorption of 5 nm TiO_2_ NPs leads to the formation of a sandwich structure in which a homogeneous distribution of the semiconductor ensures an efficient physical interaction with the plasmonic NPs (Figure 2). In order to obtain reproducible and comparable results, three different Au/TiO_2_ molar ratios were used (0.023, 0.059, and 0.1), while keeping the amount of TiO_2_ NPs constant. The analysis of the TEM images shows that the Au NRs adsorbed by electrostatic interactions onto the titanate nanowires have a preferential orientation by which they tend to follow the main axis of the template (Figure 2a–c). In this manner, more than 75% of the plasmonic objects lie within the axis of the nanowires (accepting a deviation of ±30°) (Figure 2d, for more details see the Experimental Section). This preferential deposition represents a major difference with respect to the use of spherical templates in which random distributions are observed [6]. In what concerns the optical properties of the hybrids, extinction spectra show an important contribution at high energies coming from the absorption and scattering contributions of the TiO_2_ NPs and the titanate templates, respectively. Nevertheless, the most significant signature comes from the longitudinal plasmon band of the Au NRs. Interestingly, the three samples show a red shift of this signature with respect to the free Au NRs in solution, being this effect a consequence of the optical coupling between the plasmonic objects. As expected, this red shift increases with the concentration of Au NRs, reaching an absorption maximum of the longitudinal plasmon band at 1008 nm for the highest concentration of plasmonic metal (Figure 2e, inset). The one-dimensional alignment of the Au NRs is a subject of paramount importance in this work because the directed coupling of the nanorods can lead to the formation of extremely high electromagnetic field enhancements (plasmonic hot spots) [9,15]. This effect can substantially increase the population of hot electrons that can be injected into the conduction band of a nearby semiconductor, hence increasing its photosensitization and the overall catalytic activity of the system. In order to corroborate this assumption, a control experiment was performed with spherical Au NPs instead of NRs (Figure S3b). In this case, the absorption signature due to the spherical objects was centered at 532 nm and underwent no variation with an increase in the metal concentration (Figure S3a). Such a feature demonstrates that the observed preferential 1-dimensional plasmonic coupling with the Au NRs was suppressed. Accordingly, an important decrease in the photosensitization mechanism was expected for the hybrids based on spherical Au NPs.

**Figure 3:**
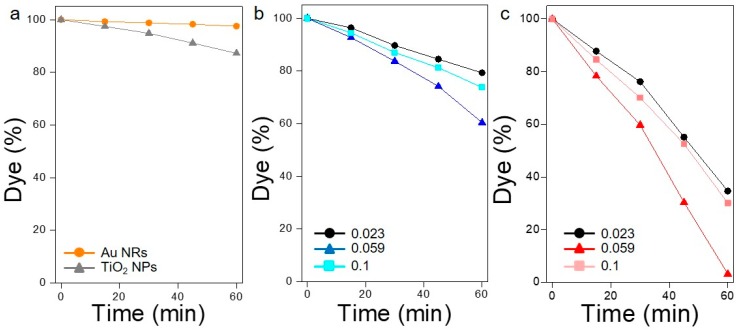
Photodegradation profiles of RhB in the presence of different photocatalysts: (**a**) reference samples consisting of Ti NWs functionalized with Au NRs (orange circles) or TiO_2_ NPs (grey triangles), separately; (**b**) Ti NWs with TiO_2_ NPs and spherical Au NPs (Au/TiO_2_ molar ratios: 0.023 in black circles, 0.059 in blue triangles and 0.1 in cyan squares); and (**c**) Ti NWs functionalized with TiO_2_ NPs and Au NRs (Au/TiO_2_ molar ratios: 0.023 in black circles, 0.059 in red triangles and 0.1 in pink squares).

The photodegradation of rhodamine B was chosen as a reliable chemical probe to measure the extent of plasmonic photosensitization of TiO_2_ in the hybrid composites. Prior to this, two control experiments were performed in order to ascertain the activity of the different components when used separately. In this regard, the deposition of TiO_2_ alone onto Ti NWs led to a fairly limited photodegradation (13%) after 1 h of irradiation with a solar simulator (Figure 3a, grey triangles). This residual activity is explained as a consequence of the small portion of radiation of the solar spectrum that has enough energy to produce an electron transition between the valence and the conduction bands of the semiconductor (UV light). Furthermore, an absence of photocatalytic activity was observed when only Au NRs are adsorbed onto the Ti NWs (without TiO_2_), thus excluding any possible thermal effect induced by the plasmonic excitation and the subsequent electron–phonon thermalization (Figure 3a, orange spheres). Alternatively, the photodegradation of RhB was only satisfactorily achieved when both components (photosensitizer and acceptor) were combined synergistically onto the support. Indeed, when Au NRs were used as active components with a metal/TiO_2_ molar ratio of 0.023, a degradation of 65% was attained after 1 h of irradiation (Figure 3c). Interestingly, an almost quantitative degradation (97%) of the organic dye was observed after the same period of time when the molar ratio was increased to 0.059. Nevertheless, further increases in the concentration of the plasmonic objects led to an important decrease in the catalytic activity. Such behavior has been previously explained as a consequence of a faster recombination kinetics for the electron-hole pair above certain metal concentrations [6,8,30]. Under these specific conditions, metal components may behave as recombination centers inducing the loss of the necessary reactive species that contribute to the photocatalytic activity of the hybrid system. It is important to point out that the photocatalysts show no degradation after the photocatalytic tests. Along these lines, TEM characterization of the objects shows no modification in the structural properties of the hybrid materials (Figure S4) after photodegradation of RhB.

In order to establish the role played by the anisotropic plasmonic coupling in the final enhancement of the photocatalytic activity, the same experiment was performed replacing the Au NRs by spheres with a diameter of 45 nm (Figure 3b). In this case, a degradation of 39.5% was observed for the optimized Au/TiO_2_ molar ratio. This important decrease in the photocatalytic activity (from 97% to 39.5%) is ascribed to the inability of the Au spheres to produce unidirectional hot spots onto the Ti NWs, thus decreasing the magnitude of the electromagnetic field enhancement at the metal–semiconductor interface. These results undoubtedly show the importance of the shape and curvature of the substrate in the selective formation of unidirectional coupling between the Au NRs.

## 4. Conclusions

In the present study, we report the use of Ti NWs (with a Na_0.915_H_1.085_Ti_3_O_7_·*n*H_2_O chemical composition) as advantageous substrates for the development of hybrid photocatalysts. In this manner, TEM, XRD and Raman were used for an in-depth characterization of the chemical and structural properties of these objects. Subsequently, the Ti NWs were functionalized with Au NRs and TiO_2_ NPs, leading to a new class of hybrid nanostructures with outstanding photocatalytic features. These extraordinary properties are explained as a consequence of the anisotropic morphology of the substrates and the preferential directed 1D assembly of the Au NRs, thus leading to the controlled formation of plasmonic hot spots. The results obtained with the proposed hybrid material illustrate that the rational assembly of different components at the nanoscale is a characteristic of paramount importance towards the development of broadband photocatalysts with enhanced activities. In summary, high aspect ratio templates are envisioned as ideal candidates for the improvement of plasmonic photocatalysis.

## Supplementary Materials

The following are available online at https://www.mdpi.com/2079-4991/9/7/990/s1. Table S1. Cell parameters for the as-synthesized sodium titanate nanowires, calculated using the Le Bail method (reliability factors: Rp = 4.793, Rwp = 6.579). * Reference values have been taken from the Crystallography Open Database (CIF files: 2310331 (Na2Ti3O7), 4000748 (Na2Ti6O13) and 2310730 (Na2Ti9O19)). Figure S1. Size histogram representing the mean diameter of the TiNWs before functionalization. Figure S2. Raman spectra of the sodium titanate nanowires registered using variable laser powers under a 532 (**a**), 633 (**b**) or 785 nm (**c**) excitation wavelength. Raman spectra collected from different spots in the same sample, using a 785 nm excitation wavelength and a laser power of 5.13 mW (**d**). For all these measurements an acquisition time of 60 s has been used. Figure S3. (**a**) Extinction spectra of the titanate nanowires functionalized with different amounts of Au spheres NPs and TiO2 NPs. The Au/TiO2 molar ratios are: 0.023 (black), 0.059 (blue) and 0.1 (cyan). (**b**) TEM image of the sample synthesized with the intermediate amount of plasmonic component. The scale bar is 200 nm. Figure S4. TEM image of TiNWs functionalized with TiO2 NPs and Au NRs after the photocatalytic degradation of RhB (Au/TiO2 molar ratio of 0.1).
