# Supplementary material for: Titanate Nanowires as One-Dimensional Hot Spot Generators for Broadband Au–TiO2 Photocatalysis
Source: Nanomaterials (Basel). 2019 Jul 9;9(7):990. doi: 10.3390/nano9070990 (PMC6669474; doi:10.3390/nano9070990)
Supplement: Supplementary file 1 [file nanomaterials-09-00990-s001.pdf]

Supplementary Materials

Titanate Nanowires as One-Dimensional Hot Spot Generators for Broadband Au–TiO_2 Photocatalysis

Yoel Negrín-Montecelo [1], Martín Testa-Anta [2], Laura Marín-Caba [1], Moisés Pérez-Lorenzo [1], Verónica Salgueiriño [2], Miguel A. Correa-Duarte [1], Miguel Comesaña-Hermo [3,*]

1 Department of Physical Chemistry, Biomedical Research Center (CINBIO), Southern Galicia Institute of Health Research (IISGS), and Biomedical Research Networking Center for Mental Health (CIBERSAM), Universidade de Vigo, 36310 Vigo, Spain
2 Departamento de Física Aplicada, Universidade de Vigo, 36310 Vigo, Spain
3 Université de Paris, ITODYS, CNRS, UMR 7086, 15 rue J-A de Baïf, F-75013 Paris, France
* Correspondence: miguel.comesana-hermo@univ-paris-diderot.fr

Table S1. Cell parameters for the as-synthesized sodium titanate nanowires, calculated using the Le Bail method (reliability factors: Rp = 4.793, Rwp = 6.579). *Reference values have been taken from the Crystallography Open Database (CIF files: 2310331 ($Na_2Ti_3O_7$), 4000748 ($Na_2Ti_6O_{13}$) and 2310730 ($Na_2Ti_9O_{19}$)).

PHASE	CELL PARAMETER	REFINED VALUE	REFERENCE VALUE*
$NA_2TI_3O_7$ (SPACE GROUP: $P2_1$/M)	A (NM)	0.8670	0.8571
	B (NM)	0.3799	0.3804
	C (NM)	0.9292	0.9135
	Β (°)	102.33	101.57
$NA_2TI_6O_{13}$ (SPACE GROUP: C2/M)	A (NM)	1.5109	1.5110
	B (NM)	0.3734	0.3744
	C (NM)	0.9367	0.9169
	Β (°)	98.75	98.98
$NA_2TI_9O_{19}$ (SPACE GROUP: C2/M)	A (NM)	1.2241	1.2200
	B (NM)	0.3758	0.3780
	C (NM)	1.5658	1.5600
	Β (°)	104.60	105.00

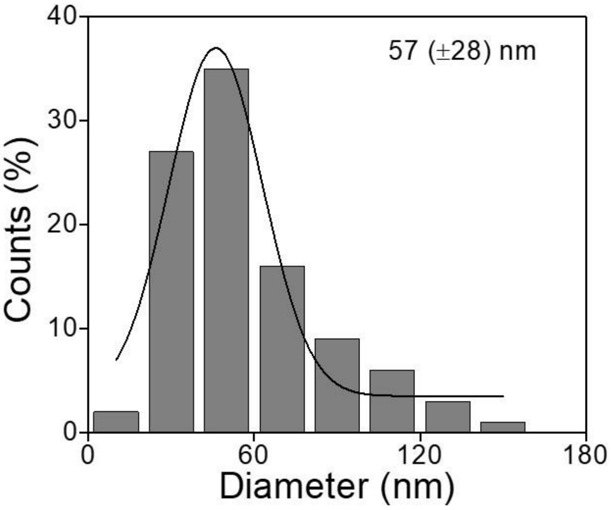

Figure S1. Size histogram representing the mean diameter of the TiNWs before functionalization.

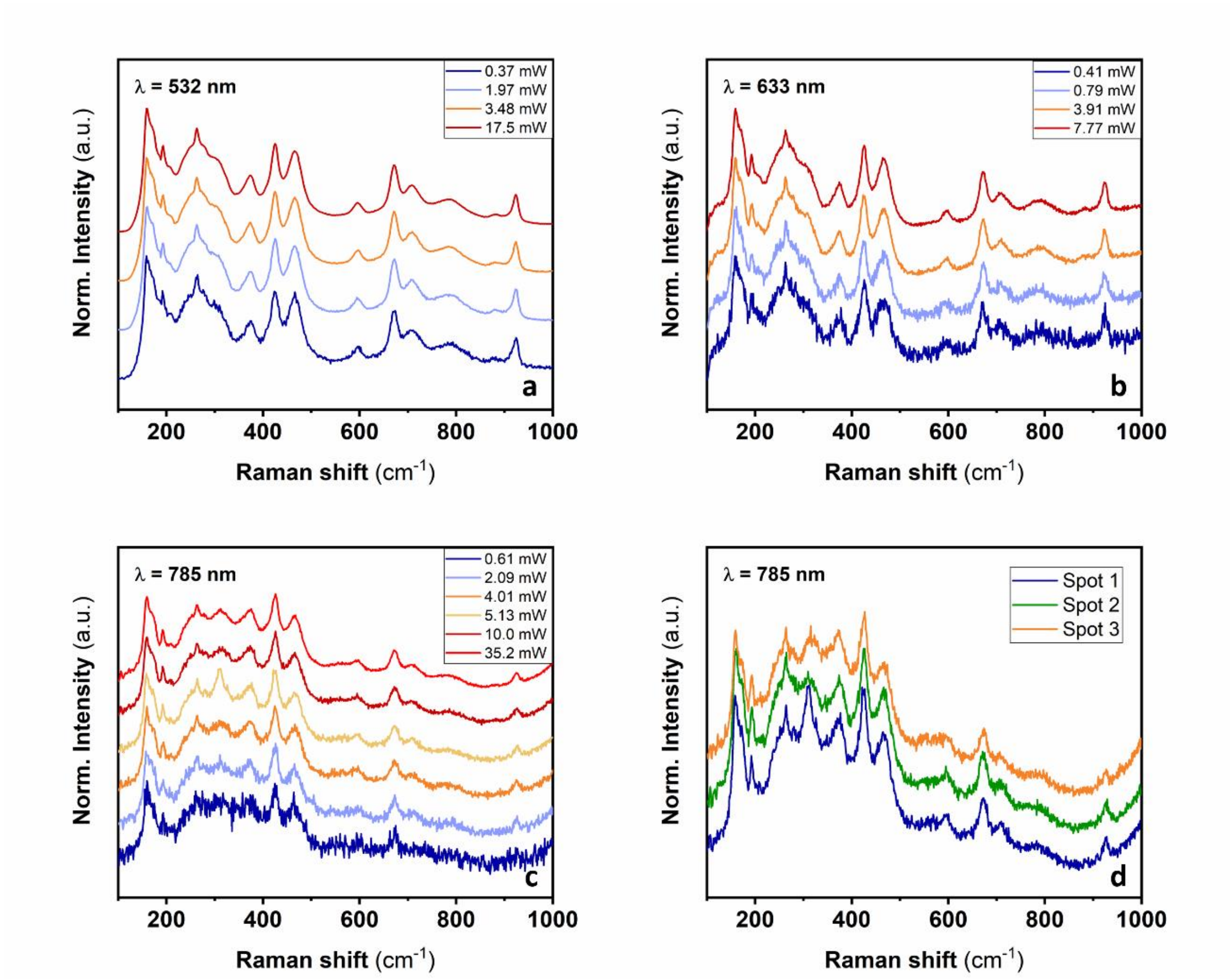

Figure S2. Raman spectra of the sodium titanate nanowires registered using variable laser powers under a 532 (**a**), 633 (**b**) or 785 nm (**c**) excitation wavelength. Raman spectra collected from different spots in the same sample, using a 785 nm excitation wavelength and a laser power of 5.13 mW (**d**). For all these measurements an acquisition time of 60 s has been used.

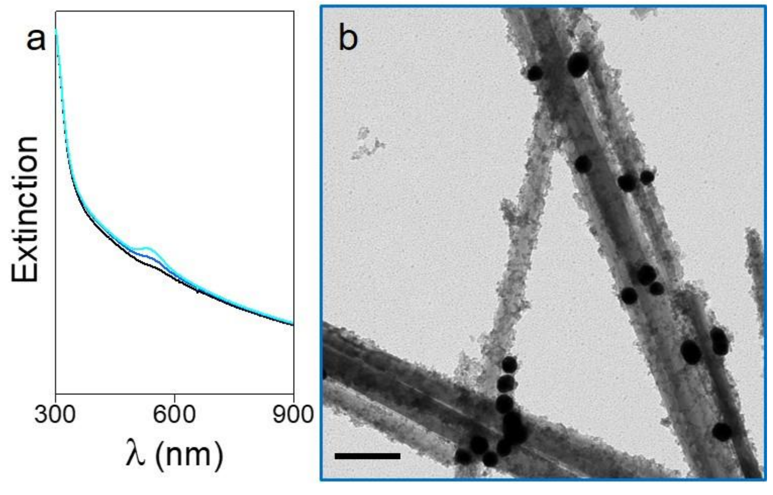

Figure S3. (**a**) Extinction spectra of the titanate nanowires functionalized with different amounts of Au spheres NPs and TiO_2 NPs. The Au/TiO_2 molar ratios are: 0.023 (black), 0.059 (blue) and 0.1 (cyan). (**b**) TEM image of the sample synthesized with the intermediate amount of plasmonic component. The scale bar is 200 nm.

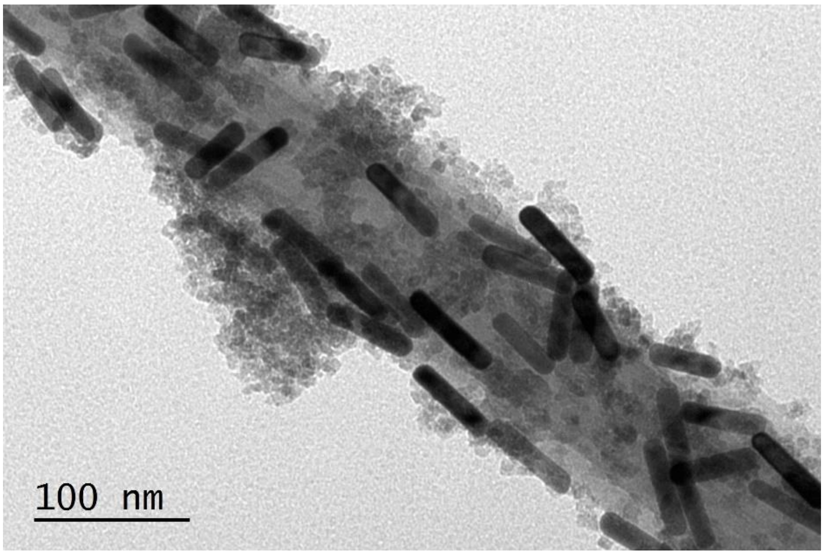

Figure S4. TEM image of TiNWs functionalized with TiO_2 NPs and Au NRs after the photocatalytic degradation of RhB (Au/TiO_2 molar ratio of 0.1).
